# Luminescence of Mn^4+^ in a Zero-Dimensional Organic–Inorganic Hybrid Phosphor [N(CH_3_)_4_]_2_ZrF_6_ for Dual-Mode Temperature Sensing

**DOI:** 10.3390/ma15196543

**Published:** 2022-09-21

**Authors:** Jing Wang, Jitao Lu, Yahong Wu, Mingjun Song

**Affiliations:** School of Chemistry and Chemical Engineering, Weifang University, Weifang 261061, China

**Keywords:** zero-dimensional, Mn^4+^, organic–inorganic hybrid

## Abstract

Searching for new low-dimensional organic–inorganic hybrid phosphors is of great significance due to their unique optical properties and wide applications in the optoelectronic field. In this work, we report a Mn^4+^ doped zero-dimensional organic–inorganic hybrid phosphor [N(CH_3_)_4_]_2_ZrF_6_, which was synthesized by a wet chemical method. The crystal structure, thermal stability, and optical properties were systemically investigated by means of XRD, SEM, TG-DTA, FTIR, DRS, emission spectra, excitation spectra, as well as decay curves. Narrow red emission with high color purity can be observed from [N(CH_3_)_4_]_2_ZrF_6_:Mn^4+^ phosphor, which maintains effective emission intensity even at room temperature, indicating its potential practical application in WLEDs. In the temperature range of 13–295 K, anti-Stokes and Stokes sidebands of Mn^4+^ ions exhibit different temperature responses. By applying the emission intensity ratio of anti-Stokes vs. Stokes sidebands as temperature readout, an optical thermometer with a maximum absolute sensitivity of 2.13% K^−1^ and relative sensitivity of 2.47% K^−1^ can be obtained. Meanwhile, the lifetime Mn^4+^ ions can also be used for temperature sensing with a maximum relative sensitivity of 0.41% K^−1^, demonstrating its potential application in optical thermometry.

## 1. Introduction

In recent years, luminescent materials have attracted special attention for their applications in optoelectronics, especially in the fields of temperature sensing [1,2,3]. Traditional thermometers are based on the expansion properties of liquids or metals, requiring contact with objects and heat exchange, thus leading to errors between the measured temperature and the actual temperature [4,5]. Therefore, temperature sensors based on luminescent materials are in high demand due to their high accuracy and non-invasive properties. Optical thermometry can be performed using a number of techniques, such as emission intensity, decay lifetime, band width, peak shift, and fluorescence intensity ratio (FIR). Among them, FIR and decay lifetime techniques have a self-reference character to remove the disturbance of excitation power or detector stability. The traditional FIR technique is based on the intensity ratio between two emissions originating from two thermally coupled energy levels of rare earth ions in up-conversion processes, such as Er^3+^ (^2^H_11/2_/^4^S_3/2_), Ho^3+^ (^5^S_2_/^5^F_4_), Tm^3+^ (^3^F_2,3_/^3^H_4_), and so on [6,7,8]. However, such phosphors have some defects, such as large overlap of emission peaks of thermally coupled energy levels, especially in the high temperature range, which reduces the measurement accuracy. Furthermore, the absorption of rare earths in the near infrared region is weak, and the quantum efficiency is low [9,10,11,12,13,14,15]. Therefore, it is necessary to develop a new optical thermometer with the advantages of high peak resolution, low cost, and effective emission.

Mn^4+^ doped fluoride phosphors have attracted much attention due to their low production cost and wide absorption from the ultraviolet to blue region [16,17]. The Mn^4+^ ion has been regarded as one of the most promising activators for narrow red emission, because its emission locates at about 630 nm in fluoride crystal fields. Meanwhile, the narrow emission of Mn^4+^ consists of some vibronic transitions due to an ^2^E_g_–^4^A_2g_ transition, which can be regarded as thermally coupled vibronic levels. Hence, Mn^4+^ doped fluoride phosphor is an excellent candidate for applications in fluorescence thermometers [18,19,20,21,22,23,24]. Recently, zero-dimensional organic inorganic phosphors have attracted unparalleled interest owing to their unique crystallographic structures with fascinating optical characteristics [25]. The organic ingredients expand the atomic distances between isolated luminescent centers, expanding the 0D family and leading to unprecedented luminescence properties. Although tunable broadband emission can be obtained from 0D metal halides, there are few reports on narrow band emission in 0D systems. Recently, T. Jüstel et al. reported narrow red emission in organic–inorganic [C(NH_2_)_3_]_2_GeF_6_:Mn^4+^ phosphor [26]. However, the narrow red emission of the Mn^4+^ in this material could only be observed at cryogenic temperature due to the multi-phonon relaxation. P. Cai et al. reported an organic–inorganic [N(CH_3_)_4_]_2_TiF_6_:Mn^4+^ phosphor, whose narrow red emission of Mn^4+^ can be observed even at room temperature [27]. Inspired by this, we synthesized organic–inorganic [N(CH_3_)_4_]_2_ZrF_6_:Mn^4+^ phosphor directionally. Accordingly, luminescence properties of 0D metal halides mainly depend on the intrinsic photophysical properties of their inorganic building blocks. Therefore, the substitution of Ti by Zr would exhibit excellent photoelectric properties.

In the present work, we adopt a wet chemical method to obtain a zero-dimensional organic–inorganic hybrid phosphor, [N(CH_3_)_4_]_2_ZrF_6_:Mn^4+^, which has been synthesized by combining hydrothermal and solid state methods. The photoluminescence property as well as the dynamic decay of [N(CH_3_)_4_]_2_ZrF_6_:Mn^4+^ phosphor have been investigated in a wide temperature range from 13 to 295 K.

## 2. Materials and Methods

### 2.1. Sample Preparation

[N(CH_3_)_4_]_2_ZrF_6_: Mn^4+^ powder samples were successfully synthesized by a wet chemical method [27]. K_2_MnF_6_ powders were used for the Mn^4+^ source and prepared according to the published literature [10,28]. Pure [N(CH_3_)_4_]_2_ZrF_6_ powder was prepared by hydrothermal method [29]. Practically, 0.493 g ZrO_2_, 0.219 g [N(CH_3_)_4_]Cl, 10.0 mL DMF, and 1.25 mL HF (48%) were mixed and stirred for 30 min in a Teflon reactor. Then, the mixture solution was heated at 180 °C for 48 h in an oven. Natural cooling to room temperature, the crystalline sample was collected by filtration. Washed with DMF for three times, the product was dried at 100 °C for 12 h. Mn^4+^ doped [N(CH_3_)_4_]_2_ZrF_6_ was obtained by facile solid-state method. Appropriate [N(CH_3_)_4_]_2_ZrF_6_ and K_2_MnF_6_ in stoichiometric (1% mol) were well weighted and ground thoroughly in an agate mortar. Then, the mixture was heated in an oven at 80 °C for 24 h.

### 2.2. Characterization

The crystalline information of the obtained products was identified by means of X-ray diffraction (XRD, Philips X’Pert MPD, Almelo, The Netherlands). The morphology was evaluated by JSM-6700F field emission scanning electron microscopy (SEM). Thermal analyses were performed on a Shimadzu (Japan) thermal analyzer with a heating rate of 10 °C/min from room temperature to 800 °C in N_2_ atmosphere (30 mL/min). Fourier transformed infrared (FTIR) spectrum was measured by using a FT/IR-4100 (JASCO) spectrometer in ATR mode with sample powder. The quantum yield (QY) of the obtained sample was measured by the Edinburgh Instruments FLS1000 equipped with an integrating sphere attachment. The concentration of Mn^4+^ in the sample was analyzed by inductively coupled plasma–mass spectrometry (ICP-MS) on a Perkin Elmer (NexION 300D). The diffuse reflectance spectrum (DRS) was recorded by a V-670 (JASCO) spectrophotometer. The photoluminescence excitation (PLE) spectrum was recorded by a QuantaMaster 300 (PTI) high power Xenon flash lamp. The photoluminescence (PL) spectra and decay lifetimes were recorded by using a 355 nm pulsed Nd:YAG laser (Spectron Laser Sys. SL802G) with a pulse width of 5 ns and a repetition rate of 10 Hz. The luminescence is collected by the 75 cm monochromator (ActonResearch Corp. Pro-750, Acton, MA, USA) and multiplied by the PMT (Hamamatsu R928, Hamamatsu Electronic Press Co., Ltd., Shizuoka, Japan). The temperature was controlled by a liquid helium flow cryostat.

## 3. Results

**Figure 1:**
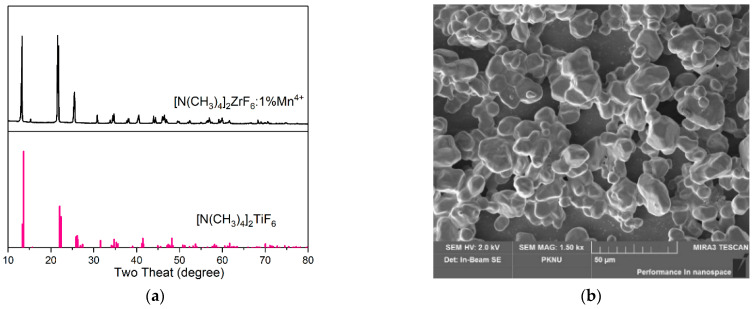
(**a**) XRD patterns of [N(CH_3_)_4_]_2_ZrF_6_:1%Mn^4+^ sample (top) at room temperature, and a standard [N(CH_3_)_4_]_2_TiF_6_ card (bottom) is given as a reference. (**b**) SEM image of [N(CH_3_)_4_]_2_ZrF_6_:1%Mn^4+^ sample.

Figure 1a shows the XRD patterns of the obtained [N(CH_3_)_4_]_2_ZrF_6_:1%Mn^4+^ sample. All the peaks match well with the standard [N(CH_3_)_4_]_2_TiF_6_ card, and no trace of impurity phase can be found [10,29], indicating that the pure [N(CH_3_)_4_]_2_ZrF_6_ phase was obtained. By the Williamson–Hall plot, the crystalline grain size of 76 nm and micro strain of 0.00467 were obtained. The morphology of the obtained phosphor is displayed in Figure 1b. The particles are in the size of ten to twenty micrometers with a smooth surface, and most of them are clustered into larger particles.

**Figure 2:**
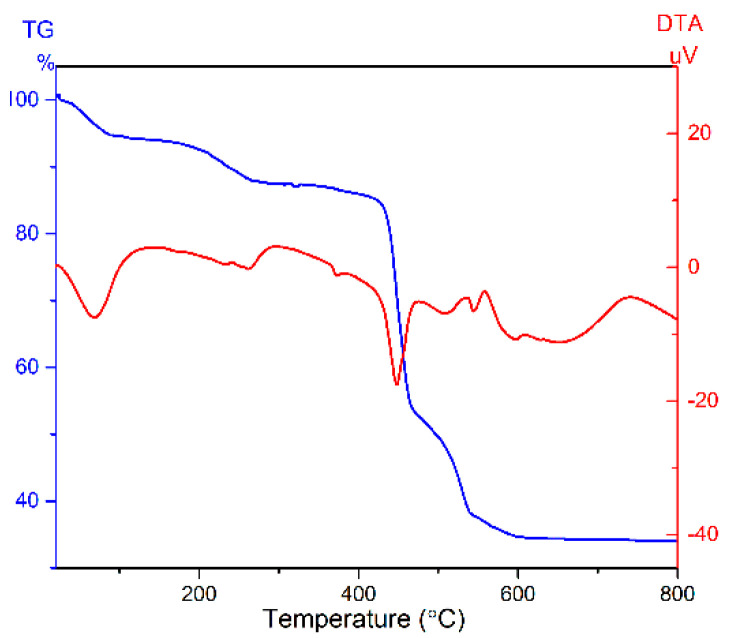
TG (blue) and DTA (red) curves of the [N(CH_3_)_4_]_2_ZrF_6_:1%Mn^4+^ sample recorded under N_2_ atmosphere.

For the purpose of confirming the thermal stability of [N(CH_3_)_4_]_2_ZrF_6_:1%Mn^4+^, TG-DTA curves were measured in N_2_ atmosphere, as shown in Figure 2. Four endothermic peaks can be observed. A broad endothermic peak at about 100 °C coincided with a 5% mass loss, which could be a loss of water adsorbed to the particle surface. The second endothermic peak occurs at 270 °C and is comparatively weak and broad, coinciding with a 10% mass loss. Since phase transition is a transition without weight loss, we tentatively assigned the peak at 270 °C as the thermal decomposition of a portion of the [N(CH_3_)_4_]^−^ group [26]. In addition, an obvious mass loss occurs at about 420 °C, coinciding with the endothermic peak, which corresponds to the decomposition of the sample and indicates a good thermal stability of [N(CH_3_)_4_]_2_ZrF_6_:1%Mn^4+^. The nature of the endothermic peak at 650 °C without mass loss is not known [27].

**Figure 3:**
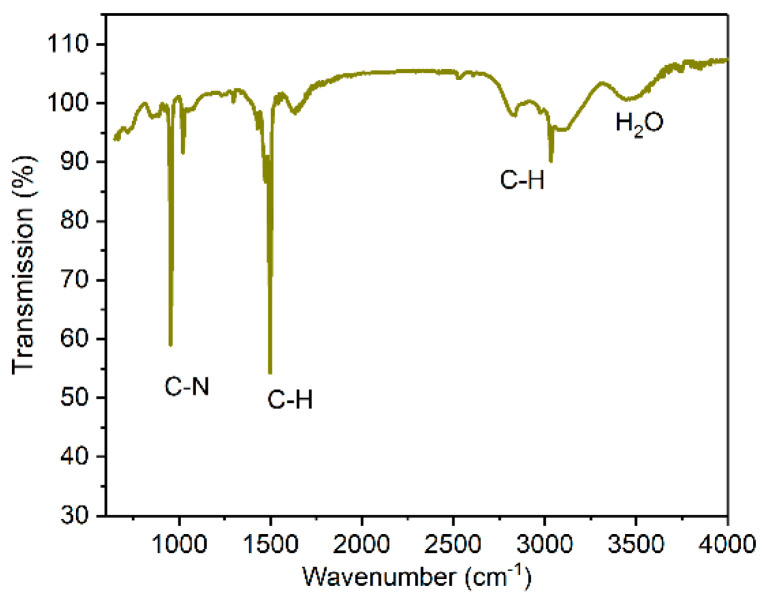
FTIR spectrum of [N(CH_3_)_4_]_2_ZrF_6_:1%Mn^4+^ sample at room temperature.

Figure 3 shows the FTIR spectrum of the synthesized [N(CH_3_)_4_]_2_ZrF_6_:1%Mn^4+^ powder. The absorption bands correspond to the vibrations of molecular groups. According to the literature, those absorption bands are marked in the spectrum. The C–N stretching vibrations can be observed at 956 and 1023 cm^−1^. The peaks located at 1472, 1598, 2830, and 3024 cm^−1^ can be ascribed to the C–H vibrations in the CH_3_ group [29,30]. The peak at 3460 cm^−1^ corresponds to the H_2_O molecular vibration [31].

**Figure 4:**
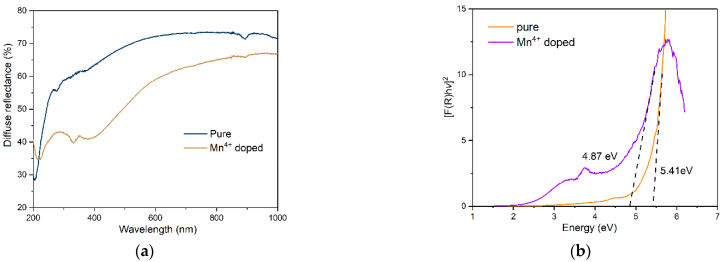
(**a**) Diffuse reflectance spectrum and (**b**) energy band gap of pure and Mn^4+^ doped [N(CH_3_)_4_]_2_ZrF_6_ samples at room temperature.

Figure 4a shows the DRS of the pure and Mn^4+^ doped [N(CH_3_)_4_]_2_ZrF_6_ samples at room temperature [32,33]. In the spectrum of undoped powder, a band at about 200 nm can be found. Upon doping with Mn^4+^, two new absorption bands at 250 and 360 nm appear, which correspond to the F^−^ (2p) → Mn^4+^ (3d) charge transfer (CT) and ^4^A_2_ → ^4^T_1_ transitions, respectively. This indicates that Mn^4+^ ions have been introduced into the host lattice successfully. In addition, the concentration of Mn^4+^ in the sample was analyzed by ICP-MS, and the concentration of Mn^4+^ (Mn:Zr = 2494:586166 μg/L) is verified to be 0.71 mol% relative to Zr. By using the Kubelka–Munk equation, the energy gap of the phosphors can be evaluated based on the transformation of DRS, as shown in Figure 4b. From the figure, the energy gap of the pure sample is about 5.41eV, while the introduction of Mn^4+^ reduces the band gap to 4.87 eV, of which the energy level of Mn^4+^ is between conduction and the valence band.

**Figure 5:**
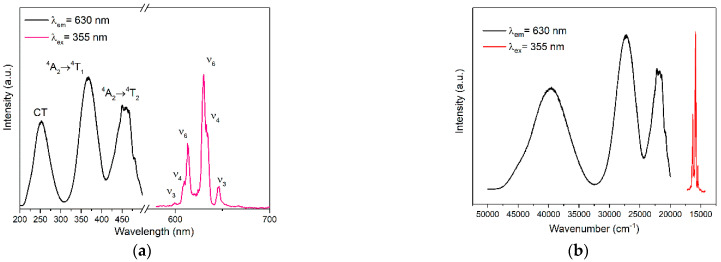
(**a**) PLE spectrum by monitoring at 630 nm, and PL spectrum with excitation of 355 nm of the [N(CH_3_)_4_]_2_ZrF_6_:1%Mn^4+^ sample at room temperature. (**b**) PLE and PL spectra with abscissa expressed in wavenumbers.

Figure 5a shows the normalized room temperature PLE and PL spectra of the as-synthesized [N(CH_3_)_4_]_2_ZrF_6_:1%Mn^4+^ phosphor. In the PLE spectrum, three excitation bands can be observed in the wavelength range from 200 to 500 nm by monitoring at 630 nm emission. The band located at 253 nm is due to the CT band, and the latter two broad bands at 360 nm (^4^A_2_ → ^4^T_1_) and 475 nm (^4^A_2_ → ^4^T_2_) correspond to the spin-allowed transitions of Mn^4+^ ions. In the PL spectrum, the narrow red emission lines can be found in the range of 580–660 nm, owing to the spin-forbidden ^2^E_g_ → ^4^A_2g_ transition of Mn^4+^ in the octahedral field. According to the research of Ok et al., [N(CH_3_)_4_]_2_TiF_6_ crystallizes in the centrosymmetric structure with zero-dimension, in which the TiF_6_ group is separated by organic cations [34]. According to S. Adachi’s summary, negligible ZPL can be found when Mn^4+^ is located in a higher crystal symmetry [1]. Thus, there is no obvious zero phonon line (ZPL) in the PL spectrum of [N(CH_3_)_4_]_2_ZrF_6_:Mn^4+^, which is similar to that of [N(CH_3_)_4_]_2_TiF_6_:Mn^4+^ [27]. However, phonon-activated sidebands can be found in either side of the ZPL. The emission peaks at 599, 609, and 613 nm can be assigned to the v_3_, v_4_, and v_6_ anti-Stokes vibrational modes, while the emission peaks at 630, 633, and 647 nm are due to the Stokes v_6_, v_4_, and v_3_ vibrational modes, respectively. Figure 5b shows the PLE and PL spectra with abscissa expressed in wavenumbers. Efficient red luminescence from [N(CH_3_)_4_]_2_ZrF_6_:Mn^4+^ phosphor could be observed by the UV light illumination, and the QY value of the sample is about 31%, as shown in Figure S1.

**Figure 6:**
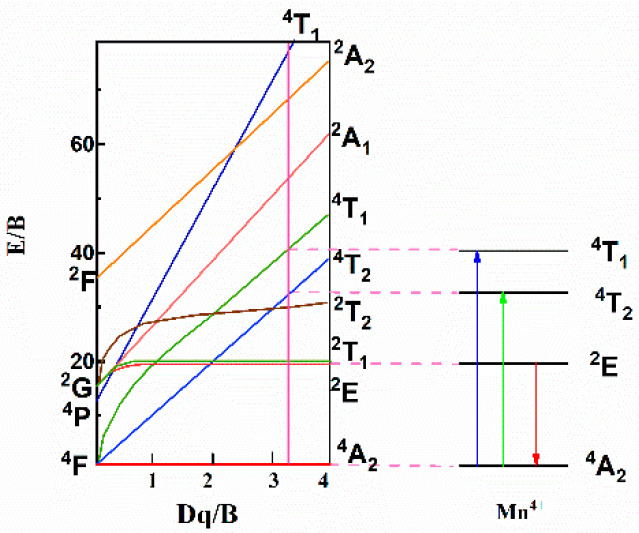
Tanabe–Sugano energy-level diagram of Mn^4+^ in [N(CH_3_)_4_]_2_ZrF_6_.

As is known, Mn^4+^ is a high field ion with 3d^3^ configuration, and its luminescence property is highly related to the crystal environment, such as crystal strength, site symmetry, covalency and nephelauxetic effect [35]. Generally, the energy level scheme of Mn^4+^ in the host can be expressed by the Tanabe–Sugano diagram [36], as shown in Figure 6. Dq stands for the local crystal field strength, which can be obtained from the mean peak energy (21,052 cm^−1^) of the ^4^*A*_2*g*_ → ^4^*T*_2*g*_ transition by using the following equation [37]:

(1) Dq=E(A42g−T42g)10

The Racah parameter *B* can be determined by the equation:

(2) $$\frac{Dq}{B}=\frac{15\left(x-8\right)}{x^{2}-10x}$$

where the parameter *x* can be defined as:

(3) x=E(A42g−T41g)−E(A42g−T42g)Dq

On the basis of the peak energy (15,873 cm^−1^) of ^2^*E_g_* → ^4^*A*_2*g*_ transition obtained from the emission spectrum, the Racah parameter *C* can be evaluated from the expression:

(4) E(E2g−A42g)B=3.05CB+7.9−1.8BDq

Based on Equations (1)–(4), the crystal field parameters of *Dq*, *B*, *C* were determined to be 2105, 638 and 3660 cm^−1^, respectively.

**Figure 7:**
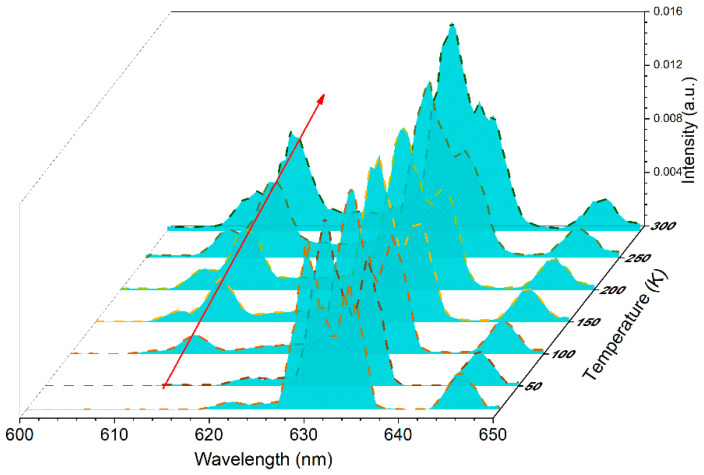
Temperature-dependent PL spectra of [N(CH_3_)_4_]_2_ZrF_6_:1%Mn^4+^ in the range from 13 to 295 K.

**Figure 8:**
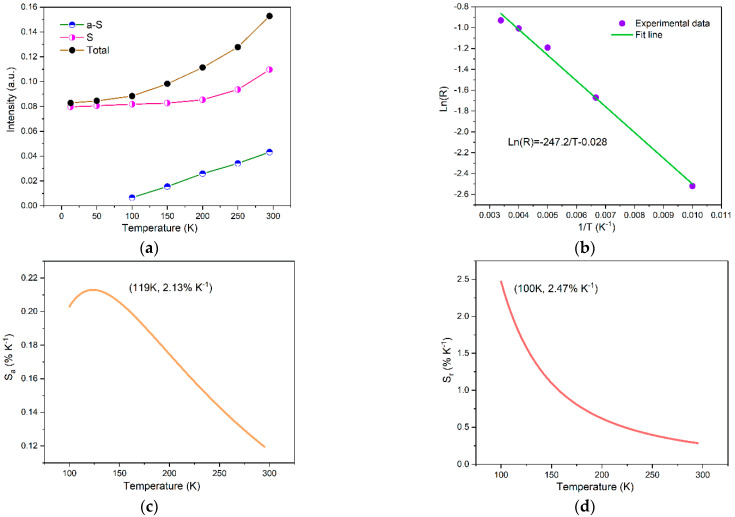
(**a**) Integral emission intensities of anti-Stokes sidebands (blue), Stokes sidebands (red), and total Mn^4+^ emission (black) as a relation with temperature from 13 to 295 K; (**b**) emission intensity ratio of anti-Stokes vs. Stokes sidebands (R) in a log relation with 1/T; (**c**) calculated absolute sensitivity; (**d**) calculated relative sensitivity.

To investigate the luminescence properties of [N(CH_3_)_4_]_2_TiF_6_:Mn^4+^ at low temperature, the temperature-dependent emission spectra were recorded in the temperature range from 13 to 295 K, as displayed in Figure 7. At 13 K, only ZPL and Stokes sidebands can be observed. With the increase in temperature, electrons have more opportunity to populate at a higher vibrational state. Therefore, anti-Stokes sidebands located at a higher energy side of ZPL start to appear at 100 K. With rising temperature, both of the emission intensities of anti-Stokes and Stokes sidebands increase. Figure 8a shows the integral emission intensities of anti-Stokes sidebands, Stokes sidebands, and total Mn^4+^ emission as a relation with temperature. It can be observed that all the emission intensities increase with increasing temperature from 100 to 295 K. According to the thermally coupled energy levels (TCL) theory of trivalent rare earth ions, the adjacent levels with energy gaps between 100–2000 cm^−1^ can be thermally populated. Therefore, the phonon-coupled anti-Stokes and Stokes sidebands could be considered as two adjacent levels. According to Boltzmann’s law, the emission intensity ratio (R) from thermally coupled energy levels can be expressed as:

(5) $$R=Aexp\left(-\frac{∆E}{kT}\right)$$

By transforming Equation (5), the emission intensity ratio (R) of anti-Stokes vs. Stokes sidebands of Mn^4+^ can be fitted by the formula:

(6) $$LnR=-\frac{A}{T}+B$$

where *A* is a constant, and *B* is a correction term for the comprehensive population of thermally coupled energy levels [38]. Figure 8b shows the experimental data fitted by using Equation (6), and the values of A and B were obtained to be 247.2 and −0.028, respectively. Sensitivity is an important parameter to evaluate the suitability for thermometry. The absolute sensitivity (S_a_) and relative sensitivity (S_r_) can be defined as:

(7) $$S_{a}=\frac{\mathrm{dR}}{\mathrm{dT}}=\frac{A}{T^{2}}e^{\frac{\mathrm{BT}-A}{T}}$$

(8) $$S_{r}=\frac{1}{R}\frac{\mathrm{dR}}{\mathrm{dT}}=\frac{A}{T^{2}}$$

Figure 8c,d exhibit the calculated sensitivities by using Equations (7) and (8). The absolute sensitivity increases first with rising temperature, reaching a maximum of 2.13% K^−1^ at 119 K and then decreasing to 0.119% K^−1^ at 295 K. As for the relative sensitivity, it decreases with increasing temperature from 2.47% K^−1^ at 100 K to 0.28% K^−1^ at 295 K. The obtained sensitivities are comparable to recently reported phosphors, Refs. [39,40] indicating its potential application in the FIR technique based on optical temperature sensing.

**Figure 9:**
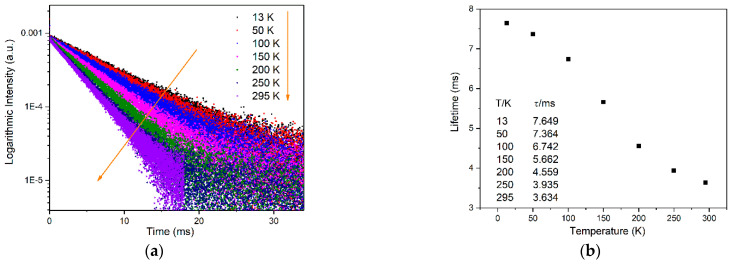

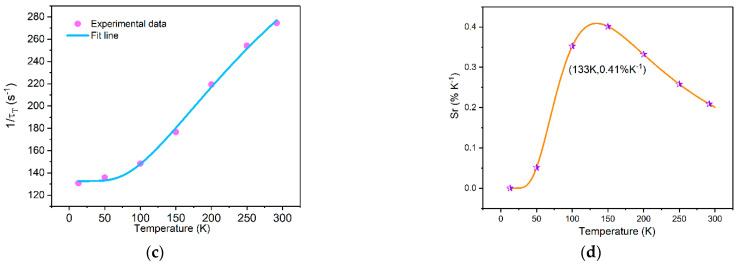
(**a**) Temperature-dependent decay curves in the temperature range from 13 to 295 K; (**b**) calculated lifetime values; (**c**) lifetime values as a relation with temperature and the fitting line; (**d**) calculated relative sensitivities.

However, the dynamic lifetime decay of the emission shows different temperature dependence from emission intensity. Figure 9a shows the decay curves of a Mn^4+^ doped [N(CH_3_)_4_]_2_ZrF_6_ phosphor in the temperature range from 13 to 295 K. With increasing temperature, the decay curves always keep single exponential characteristics, and lifetimes become shorter, indicating the enhancement of a non-radiative relaxation process with rising temperature. According to [1,27,41,42], the excited dynamic of Mn^4+^ in fluoride phosphors is commonly related to activation energy, spin-orbit interaction and vibration modes. Therefore, the kinetic process of the ^2^E_g_ → ^4^A_2g_ emission was complicated in the whole measured temperature range, and it is difficult to establish a simple non-radiative transition theoretical model to describe the temperature-dependent decay behavior of Mn^4+^ doped phosphors. In this case, the average lifetime was adopted to evaluate the values, which can be calculated by the equation [43]:

(9) $$I\left(t\right)=A_{1}\exp\left(-\frac{t}{\tau_{1}}\right)+A_{2}\exp\left(-\frac{t}{\tau_{2}}\right)$$

(10) $$\tau_{ave}=\frac{A_{1}\times \tau_{1}^{2}+A_{2}\times \tau_{2}^{2}}{A_{1}\times \tau_{1}+A_{2}\times \tau_{2}}$$

where *I(t)* is the intensity at time *t*, *A*_1_ and *A*_2_ represent the pre-exponential factors, *τ*_1_ and *τ*_2_ stand for the fast decay lifetime and slow decay lifetime, respectively, and *τ_ave_* is the average lifetime. The calculated lifetime values at various temperatures are listed in Figure 9b. The values decrease from 7.649 to 3.634 ms as temperature increases from 13 to 295 K.

For practicality and conciseness, the temperature dependence of the decay lifetime can be well fitted based on the Mott−Seitz model, as follows [44]:

(11) $$\frac{\tau_{0}}{\tau_{T}}=1+A^{\prime }\exp\left(\frac{-E_{a}}{k_{B}T}\right)$$

where *τ*_0_ is the decay lifetime at 13 K, *τ_T_* is the decay lifetime at temperature *T*, *A*′ is an empirical pre-exponential factor, *E*_a_ is the activation energy, and *k_B_* is the Boltzmann constant. Here, in order to be more practical, *τ*_0_ is regarded as a constant, and the fitting equation is transformed to:

(12) $$\frac{1}{\tau_{T}}=B^{\prime }+C^{\prime }\exp\left(\frac{D^{\prime }}{T}\right)$$

where *B*′, *C*′, and *D*′ are the empirical parameters related to τ_0_, *A*′ and *E_a_*. After fitting the experimental data by using Equation (12) (Figure 9c), the values of *B*′, *C*′, and *D*′ are determined to be 132.7, 465.7, and −342.0, respectively. Therefore, the relative sensitivity (*S_r_*) can be calculated from:

(13) $$S_{r}=\left|\frac{\partial \tau }{\tau \partial T}\right|\times 100\%=\frac{D^{\prime }}{T^{2}}\tau C^{\prime }\exp\left(\frac{D^{\prime }}{T}\right)\times 100\%$$

The obtained *S_r_* is shown in Figure 9d, which increases firstly with rising temperature, reaching a maximum of 0.41% K^−1^ at 133 K, and then decreasing. Relying on the effective FIR technique in the temperature range from 100 to 295 K and lifetime thermometry in the temperature range from 13 to 295 K, the unique application potential of [N(CH_3_)_4_]_2_ZrF_6_:1%Mn^4+^ phosphor in the low temperature range can be forecasted.

## 4. Conclusions

A novel Mn^4+^ doped zero-dimensional organic–inorganic hybrid [N(CH_3_)_4_]_2_ZrF_6_ phosphor was successfully synthesized by a wet chemical method for the first time. The photoluminescence from the [N(CH_3_)_4_]_2_ZrF_6_:Mn^4+^ phosphor exhibits sharp lines and can be observed even at room temperature. The phosphor can be effectively excited by NUV and blue light. Temperature-dependent emission spectra and decay curves were investigated to clarify the enhanced emission intensity and the shortened lifetime, which are due to the phonon-assistant emission and enhanced non-radiative transition during the temperature-raising process. The emission intensity ratio of anti-Stokes and Stokes sidebands can be used for temperature sensing with a maximum absolute sensitivity of 2.13% K^−1^ and relative sensitivity of 2.47% K^−1^. In addition, the decay lifetime of Mn^4+^ can be used as a thermometry, demonstrating its potential application in a dual-mode temperature sensor.

## Data Availability

The data presented in this study are available from the corresponding authors upon reasonable request.

## Supplementary Materials

The following supporting information can be downloaded at: https://www.mdpi.com/article/10.3390/ma15196543/s1, Figure S1. The quantum yield of the [N(CH_3_)_4_]_2_ZrF_6_:1%Mn^4+^ sample. Figure S2. The fitting of temperature-dependent decay curves. The decay curves are recorded by monitoring at 630 nm with excitation at (a) 13 K, (b) 50 K, (c) 100 K, (d) 150 K, (e) 200 K, (f) 250 K, and (g) 295 K.
