# Supplementary material for: Luminescence of Mn4+ in a Zero-Dimensional Organic–Inorganic Hybrid Phosphor [N(CH3)4]2ZrF6 for Dual-Mode Temperature Sensing
Source: Materials (Basel). 2022 Sep 21;15(19):6543. doi: 10.3390/ma15196543 (PMC9570700; doi:10.3390/ma15196543)
Supplement: Supplementary file 1 [file materials-15-06543-s001.zip › materials-1907455-supplementary.pdf]

*materials*

*Article*

# Luminescence of $Mn^{4+}$ in a Zero-Dimensional Organic–Inorganic Hybrid Phosphor $[N(CH_3)_4]_2ZrF_6$ for Dual-Mode Temperature Sensing

**Jing Wang, Jitao Lu, Yahong Wu and Mingjun Song ***

School of Chemistry and Chemical Engineering, Weifang University, Weifang 261061, China
* Correspondence: smj521209@126.com

**Abstract:** Searching for new low-dimensional organic–inorganic hybrid phosphors is of great significance due to their unique optical properties and wide applications in the optoelectronic field. In this work, we report a $Mn^{4+}$ doped zero-dimensional organic–inorganic hybrid phosphor $[N(CH_3)_4]_2ZrF_6$, which was synthesized by a wet chemical method. The crystal structure, thermal stability, and optical properties were systemically investigated by means of XRD, SEM, TG-DTA, FTIR, DRS, emission spectra, excitation spectra, as well as decay curves. Narrow red emission with high color purity can be observed from $[N(CH_3)_4]_2ZrF_6:Mn^{4+}$ phosphor, which maintains effective emission intensity even at room temperature, indicating its potential practical application in WLEDs. In the temperature range of 13–295 K, anti-Stokes and Stokes sidebands of $Mn^{4+}$ ions exhibit different temperature responses. By applying the emission intensity ratio of anti-Stokes vs. Stokes sidebands as temperature readout, an optical thermometer with a maximum absolute sensitivity of 2.13% $K^{-1}$ and relative sensitivity of 2.47% $K^{-1}$ can be obtained. Meanwhile, the lifetime $Mn^{4+}$ ions can also be used for temperature sensing with a maximum relative sensitivity of 0.41%$K^{-1}$, demonstrating its potential application in optical thermometry.

**Keywords:** zero-dimensional; $Mn^{4+}$; organic–inorganic hybrid

**Citation:** Wang, J.; Lu, J.; Wu, Y.; Song, M. Luminescence of $Mn^{4+}$ in a Zero-Dimensional Organic–Inorganic Hybrid Phosphor $[N(CH_3)_4]_2ZrF_6$ for Dual-Mode Temperature Sensing. *Materials* **2022**, *15*, 6543. https://doi.org/10.3390/ma15196543

Academic Editor: Toma Stoica

Received: 25 August 2022
Accepted: 17 September 2022
Published: 21 September 2022

**Publisher's Note:** MDPI stays neutral with regard to jurisdictional claims in published maps and institutional affiliations.

**Copyright:** © 2022 by the authors. Submitted for possible open access publication under the terms and conditions of the Creative Commons Attribution (CC BY) license (https://creativecommons.org/licenses/by/4.0/).

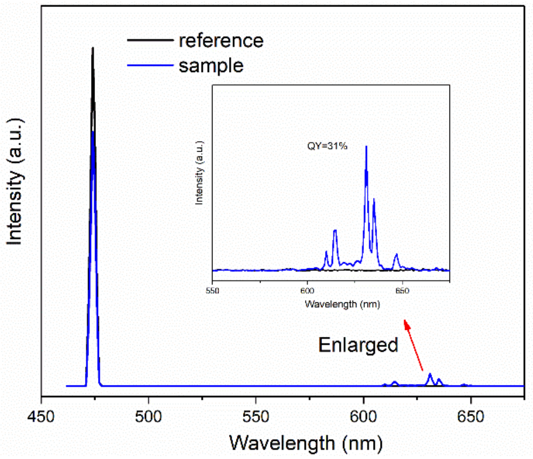


**Figure S1.** The quantum yield of the $[N(CH_3)_4]_2ZrF_6$:1%$Mn^{4+}$ sample.

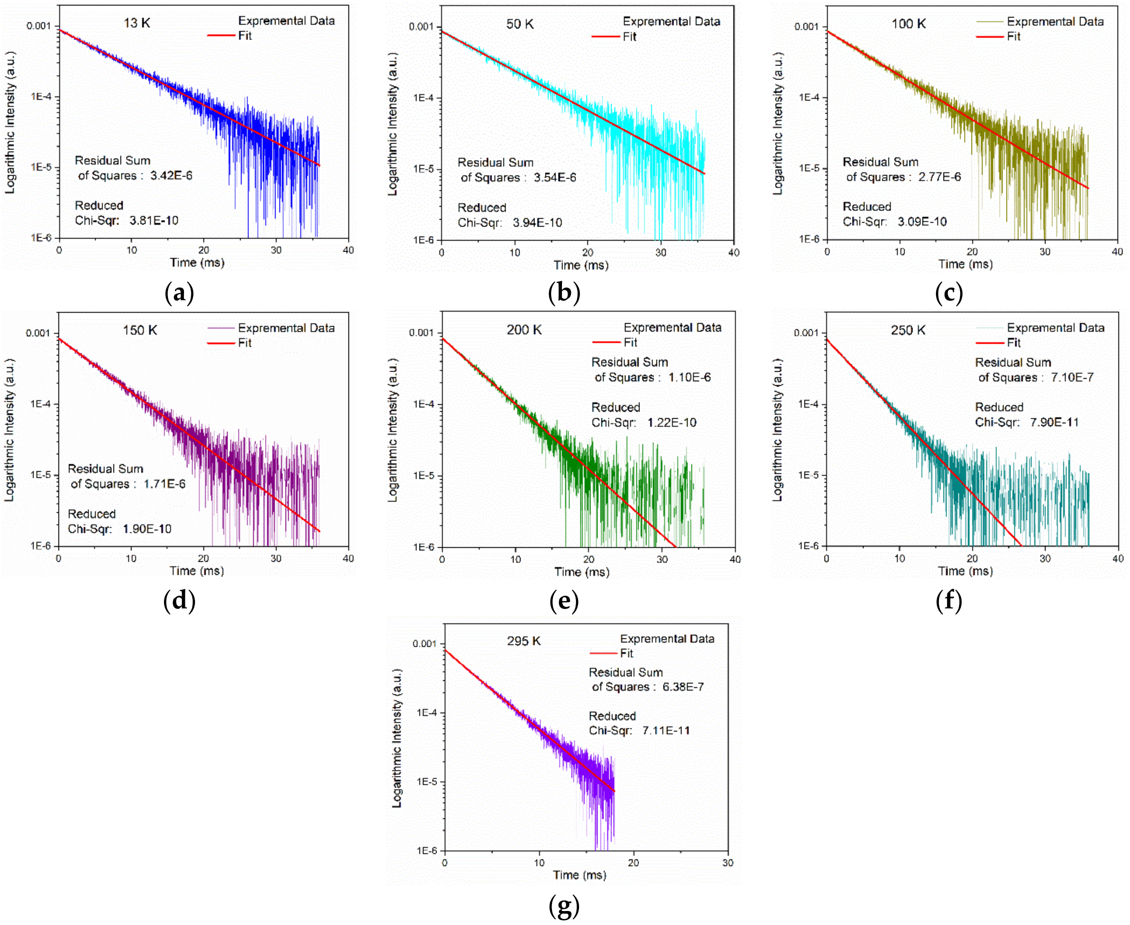


**Figure S2.** The fitting of temperature-dependent decay curves. The decay curves are recorded by monitoring at 630 nm with excitation at (a) 13 K, (b) 50 K, (c) 100 K, (d) 150 K, (e) 200 K, (f) 250 K, and (g) 295 K.
